# Serological Markers for Inflammatory Bowel Disease in AIDS Patients with Evidence of Microbial Translocation

**DOI:** 10.1371/journal.pone.0015533

**Published:** 2010-11-15

**Authors:** Anupa Kamat, Petronela Ancuta, Richard S. Blumberg, Dana Gabuzda

**Affiliations:** 1 Department of Cancer Immunology and AIDS, Dana Farber Cancer Institute, Harvard Medical School, Boston, Massachusetts, United States of America; 2 Departement de Microbiologie et Immunologie, Centre de Recherche du Centre Hospitalier de l'Universite de Montreal (CRCHUM) Universite de Montreal and INSERM Unit 743, Montreal, Quebec, Canada; 3 Department of Medicine, Brigham and Women's Hospital, Harvard Medical School, Boston, Massachusetts, United States of America; New York University, United States of America

## Abstract

**Background:**

Breakdown of the gut mucosal barrier during chronic HIV infection allows translocation of bacterial products such as lipopolysaccharides (LPS) from the gut into the circulation. Microbial translocation also occurs in inflammatory bowel disease (IBD). IBD serological markers are useful in the diagnosis of IBD and to differentiate between Crohn's disease (CD) and ulcerative colitis (UC). Here, we evaluate detection of IBD serological markers in HIV-infected patients with advanced disease and their relationship to HIV disease markers.

**Methods:**

IBD serological markers (ASCA, pANCA, anti-OmpC, and anti-CBir1) were measured by ELISA in plasma from AIDS patients (n = 26) with low CD4 counts (<300 cells/µl) and high plasma LPS levels, and results correlated with clinical data. For meta-analysis, relevant data were abstracted from 20 articles.

**Results:**

IBD serological markers were detected in approximately 65% of AIDS patients with evidence of microbial translocation. An antibody pattern consistent with IBD was detected in 46%; of these, 75% had a CD-like pattern. Meta-analysis of data from 20 published studies on IBD serological markers in CD, UC, and non-IBD control subjects indicated that IBD serological markers are detected more frequently in AIDS patients than in non-IBD disease controls and healthy controls, but less frequently than in CD patients. There was no association between IBD serological markers and HIV disease markers (plasma viral load and CD4 counts) in the study cohort.

**Conclusions:**

IBD serological markers may provide a non-invasive approach to monitor HIV-related inflammatory gut disease. Further studies to investigate their clinical significance in HIV-infected individuals are warranted.

## Introduction

CD4 T-cells in gut-associated lymphoid tissue (GALT) are primary target cells of HIV, and GALT is an important site for HIV replication and pathogenesis [1]–[4]. Significant CD4 T cell loss occurs in the gastrointestinal tract within the first few weeks of infection [5]–[7]. Later on, during chronic HIV infection, loss of GALT integrity and breaching of the gut mucosal barrier leads to microbial translocation [8]–[10], which is evidenced by the release of microbial products such as bacterial endotoxins (lipopolysaccharides (LPS), a component of gram negative bacteria) into the circulation (endotoxemia) [11]. Microbial translocation from a leaky gut may contribute to immune activation during chronic HIV infection [11]–[15]. Elevated levels of plasma LPS and bacterial 16S rDNA have been used to measure translocation of microbial products from the gastrointestinal tract [11], [16].

Microbial translocation is also a noted feature in inflammatory bowel disease (IBD) [17]–[20]. IBD comprises a group of intestinal diseases characterized by chronic inflammation of the bowel; Crohn's disease (CD) and ulcerative colitis (UC) are the common clinical subtypes of IBD. The intestinal bacterial flora triggers and drives an aberrant immune response in a genetically susceptible host, resulting in chronic inflammation of the gut [21], [22]. Low level endotoxemia has pathogenic significance in IBD; it occurs in 31%–48% of CD patients and 17%–28% of UC patients [18], [23], with a higher incidence in patients with active IBD (94% in CD, 88% in UC) [19].

The role of enteric microflora in IBD pathophysiology is highlighted by the presence of antibody reactivity to microbial antigens. These serological markers can be helpful to distinguish between CD and UC as well as aiding in the diagnosis of IBD along with clinical history, endoscopy, and physical examination [24]. The currently available IBD serological markers are: ASCA (anti-*Saccharomyces cerevisiae* antibody) [25]–[31], pANCA (perinuclear anti-neutrophil antibody) [25]–[30], [32], and anti-OmpC (antibody against outer membrane porin C of *E.coli*) [28], [33]–[37]. ASCA, which is directed against a mannose epitope in the phosphopeptidomannan of the *Saccharomyces cerevisiae* cell wall [30]–[32], is associated with CD [25]–[27], [37]–[41]. The sensitivity and specificity for detection of ASCA antibodies in CD are 50%–70% and 80%–90%, respectively [25], [27], [29], [30], [32], [37], [42], [43]. pANCA is an antibody directed against cytoplasmic constituents of neutrophils with a perinuclear staining pattern [25], [44], [45]. pANCA is detected in 40%–80% of UC patients, but also in 6%–20% of CD patients who can be classified as having a UC-like phenotype [30], [32], [37], [46], [47], with sensitivity and specificity between 55%–70% and 80%–95% in UC patients [27], [29], [30], [32], [37], [43], [45]–[47]. Anti-OmpC antibodies are detected in 40%–55% of CD patients [25], [48]–[51], with sensitivity and specificity ranging between 20%–55% and 75%–95%, respectively [25], [34], [37], [50], [52]. Anti-CBir1 (antibodies against bacterial flagellin) is a new serological marker associated with IBD [28], [35], [53]-[58]. CBir1 flagellin is an immunodominant and colitogenic antigen of enteric microbial flora [58]. Anti-CBir1 is detected in approximately 50% of CD patients [28], [35], [53], [55]–[58], and is independently associated with complicated CD [25], [33], [35], [59].

A number of parallels are observed between HIV and IBD in terms of gut disease [60]–[62] **.** Small intestinal villous atrophy and enterocyte defects have been described in HIV-infected patients. Because these defects usually occur in the absence of enteric pathogens, the term HIV enteropathy has been used [60], [62], [63]. Characteristic features of HIV enteropathy are diarrhea, gastrointestinal inflammation, increased intestinal permeability, and decreased mucosal repair and regeneration [1], [2], [60], [64]. Previous studies reported infrequent cases of IBD in HIV-infected patients [61], [65]–[67]. To our knowledge, however, no reports have examined the prevalence of ASCA, OmpC, and CBir1 antibodies in HIV infection.

We previously demonstrated that elevated circulating LPS levels correlate with monocyte activation during HIV infection, and may thereby contribute to chronic immune activation [12]. In the present study, we evaluated detection of IBD serological markers in AIDS patients with low CD4 counts (<300 cells/µl) and high plasma LPS levels. We detected IBD serological antibodies in approximately 65% of subjects. A serological pattern consistent with IBD was detected in 46% of AIDS patients; of these, 75% showed a CD-like pattern, while 25% had a UC pattern. IBD serological markers may provide a non-invasive approach to monitor HIV-related gut disease. Further studies to determine their prognostic significance in HIV-infected individuals are warranted.

## Methods

### Subjects

AIDS patients with CD4 counts <300 cells/µl were recruited at the Lemuel Shattuck Hospital (n = 20), or at 3 sites in the National NeuroAIDS Tissue Consortium (NNTC) (Manhattan HIV Brain Bank, National Neurological AIDS Bank, Texas NeuroAIDS Research Center) (n = 6) with written informed consent and IRB approval at each study site. Patients with active bacterial or opportunistic infections were excluded. There was no available radiographic, endoscopic, or histopathologic data for the gut. All plasma samples were stored at −80°C until analyzed.

### Laboratory assays

Frozen plasma samples were shipped on dry ice to Prometheus Laboratories (San Diego, CA) and analyzed in a blinded fashion; Prometheus staff did not have access to clinical information except for HIV/HCV status. Prometheus IBD Serology 7 is the most comprehensive IBD test available, utilizing several proprietary markers and incorporating computer-based Smart Diagnostic Algorithm pattern recognition technology to aid in the diagnosis of IBD as well as differentiate between CD and UC. Prometheus IBD Serology 7 tests include the following assays: ASCA IgA and IgG, anti-OmpC IgA antibodies, IgG anti-CBir1, and pANCA autoantibody by ELISA, and immunofluorescence assay (IFA) to determine the perinuclear pattern of neutrophils and DNAse sensitivity. The reference cut-off for each assay was defined by Prometheus laboratories (ASCA IgA <20 EU/ml, ASCA IgG <40 EU/ml, anti-OmpC <16.5 EU/ml, anti-CBir1 <21 EU/ml, and pANCA autoantibody <12.1 EU/ml). Positive samples showing patterns consistent with IBD were reported based on analysis by Prometheus Smart Diagnostic Algorithm Technology.

### Quantification of cytokines and chemokines

A multiplex immunoassay (Bio-source 25-plex Human Cytokine Assay; Invitrogen., CA, USA), consisting of fluorescent microspheres conjugated with a monoclonal antibody specific for a target protein, was used according to the manufacturer's instructions to measure levels of the following cytokines IL-1*β*, IL-2, IL-4, IL-5, IL-6, IL-7, IL-8, IL-10, IL-12, IL-13, IL-17, IFN-α, IFNγ, TNF-α granulocyte-monocyte colony stimulating factor (GM-CSF), monocyte chemoattractive protein (MCP-1/CCL2), macrophage inflammatory protein (MIP-1*β*/MIP-1α), IP-10, MIG, Eotaxin and RANTES. Briefly, plasma was diluted 1∶2 and incubated with antibody-coupled beads. Complexes were washed and then incubated with biotinylated detection antibody followed by streptavidin-phycoerythrin prior to assessing titers of cytokine concentration. Recombinant cytokines were used to establish standard curves. Cytokine levels were determined using a multiplex array reader from Luminex™ Instrumentation System (Bio-Plex Workstation from Bio-Rad Laboratories, USA). Analyte concentration was calculated using Bioplex Manager Software.

### Statistical analysis

Data were analyzed using the Mann-Whitney U test and Spearman rank correlation coefficient. Differences were considered significant at p<0.05.

## Results

### IBD serological markers in AIDS

To investigate the frequency and pattern of IBD serological markers in AIDS patients, we used the Prometheus IBD Serology 7 test to detect IBD serological markers in plasma samples. Subjects were selected from a larger cohort of 119 AIDS subjects with CD4 counts <300 cells/µl described in a previous study [12] on the basis of high plasma LPS above the median value for AIDS patients in the study cohort (>80 pg/ml) levels; LPS levels were determined in the previous study using the Diazo-coupled *Limulus* amebocyte lysate (LAL) assay. The study cohort consisted of 26 AIDS patients with relatively high plasma viral loads (median 10,935 copies/ml, range <50–2,210,000) and low CD4 counts (median 80 cells/µl, range 3–261), together with a high frequency of intravenous drug abuse (IVDU) (65%), HCV co-infection (50%), and HIV-associated dementia (HAD) (50%). Eleven subjects were both IVDU and HCV positive. Demographic and clinical characteristics of the study cohort are shown in Table 1. All subjects were on HAART, but only 23% were virologically suppressed (<400 plasma HIV RNA copies/ml).

**Table 1:** Demographic and clinical characteristics of HIV patients in the study cohort (n = 26).

**Age (years)**	
Median (range)	45 (32–63)
**Gender**	
Male	19 (73%)
Female	7 (27%)
**Race/ethnicity**	
African American	6 (23%)
Caucasian	12 (46%)
Hispanic	8 (31%)
**Plasma HIV RNA (copies/ml)**	
Mean ± SD	215,718±590,409
Median (range)	10,935 (<50–2,210,000)
>400 copies/ml	18 (69%)
<400 copies/ml	6 (23%)
Unknown	2 (8%)
**CD4 T Cell Count (cells/µl)**	
Mean ± SD	98.6±84.4
Median (range)	80 (3–261)
**Plasma LPS (pg/ml)**	
Mean± SD	128.07±56.18
Median (range)	109 (82.5–279.6)
**HCV Co-infection**	
Negative	7 (27%)
Positive	13 (50%)
Unknown	6 (23%)
**Substance abuse**	
No IVDU	9 (35%)
Heroin IVDU	8 (31%)
Heroin and Cocaine IVDU	5 (19%)
Cocaine IVDU	4 (15%)

In the study cohort (n = 26), ASCA IgA, pANCA, and anti-OmpC were detected in 31% (8/26), while ASCA IgG and anti-CBir1 were detected in 15% (4/26) (Table 2). Prometheus laboratories classified subjects as having a CD- or UC-like pattern according to Smart Diagnostic Algorithm Technology. According to this classification, 46% (12/26) of the subjects showed a pattern consistent with IBD, with 75% (9/12) having a CD-like pattern and 25% (3/12) having a UC-like pattern. In subjects with a positive IBD pattern, ASCA IgA and IgG antibodies were detected in 50% and 33%, while anti-OmpC, anti-CBir1, and pANCA were detected in 67%, 33%, and 42%, respectively (Table 2). Three patients with a pANCA (+) anti-OmpC (+) pattern and 1 with a pANCA (+) anti-CBir1 (+) pattern (n = 1) could also be classified as having a pattern consistent with UC-like CD [25], [29], [48], [68], [69], [70]. Thus, IBD serological markers were detected in approximately 65% of AIDS patients with high plasma LPS levels, with 46% having an IBD-like pattern and the majority of these having a CD-like pattern.

**Table 2:** Profile of inflammatory bowel disease (IBD) serological markers in AIDS subjects.

	HIV positive subjectsn = 26n (%)	Smart Algorithm positive subjectsn = 12n (%)	Heroin IVDU subjectsn = 13n (%)	HCV positive subjectsn = 13n (%)
ASCA IgA	8 (31)	6 (50)	4 (31)	5 (38)
ASCA IgG	4 (15)	4 (33)	2 (15)	2 (15)
Anti-OmpC IgA	8 (31)	8 (67)	4 (31)	3 (23)
Anti-CBir1	4 (15)	4 (33)	2 (15)	2 (15)
**NSNA (IBD Specific pANCA)**				
pANCA Autoantibody	8 (31)	5 (42)	5 (38)	2 (15)
IFA Perinuclear Pattern	5 (19)	4 (33)	2 (15)	2 (15)
DNAse Sensitivity	5 (19)	4 (33)	2 (15)	2 (15)
Crohn's- like pattern*	9 (35)	9 (75)	5 (38)	4 (31)
UC-like pattern*	3 (11)	3 (25)	1(7.6)	1 (7.6)
**UC-like Crohn's pattern**				
ANCA (+) OmpC (+)**	3 (11)	3 (11)	1 (7.6)	1 (7.6)
ANCA (+) CBir (+)***	1 (3.8)	1 (3.8)	1 (7.6)	0 (0)

Abbreviations: Heroin IVDU - Intravenous drug users using heroin, or heroin and cocaine; ASCA IgA/Ig - Anti-Saccharomyces cerevisiae antibodies; Anti-OmpC IgA- Anti-Outer membrane porin C on E.coli; Anti-CBir1- recognizes bacterial flagellin antigen associated with IBD; pANCA autontibody - IBD- specific pANCA autoantibody (NSNA, Neutrophil-specific nuclear autoantibody);

*Based on Prometheus Smart Algorithm;

**patients with UC-like pattern according to Smart Algorithm also classified as having a UC-like CD pattern;

***One patient with CD-like pattern according to Prometheus Smart Algorithm also classified as having UC-like CD pattern.

### IBD serological markers in subgroups with IV heroin use, HCV co-infection, or HIV-associated dementia (HAD)

Previous studies reported that AIDS patients with heroin IVDU, HCV co-infection or HAD have higher plasma LPS levels compared to respective control groups [12], [71]. Furthermore, substance abuse and HCV co-infection are associated with an increased incidence of bacterial infections [72]. We therefore examined the pattern of ASCA, pANCA, anti-OmpC, and anti-CBir1 in AIDS patients classified according to these subgroups (Table 2 and data not shown). There was no significant difference in the frequency or magnitude of IBD serological markers between IVDU heroin users compared to patients with no substance abuse, HCV-positive compared to HCV-negative subjects, or HAD compared to non-HAD subjects (Table 2 and data not shown). Thus, there was no difference in magnitude or frequency of IBD serological markers in these clinical subgroups.

### Higher ASCA IgG, anti-OmpC, and anti-CBir1 levels but similar plasma viral load and CD4 counts in AIDS patients with IBD-like serological pattern

Higher levels of ASCA IgG (median 31.4 EU/ml, range 12–120 compared to 12 EU/ml, 12–27.9, p = 0.027), anti-OmpC (25.9 EU/ml, 4.7–111, compared to 7.1 EU/ml, 1–15.3, p = 0.005), and anti-CBir1 (18.6 EU/ml, 8–37.7, compared to 9.1 EU/ml, 5.5–15.6, p = 0.001) were detected in subjects with a positive IBD pattern as compared to those with a negative pattern (Table 3 and Figure S1). In contrast, there was no significant difference in ASCA IgA and pANCA antibody levels between these two groups (p = 0.135 and p = 0.540). We then investigated the relationship of IBD serological markers to HIV disease markers, plasma LPS, and EndoCAb levels. HIV RNA levels, CD4 cell counts, plasma sCD14, plasma LPS, and IgM EndoCAb levels were similar in the 2 groups (Table 3). Furthermore, there were no significant correlations (Spearman correlation) between levels of individual IBD serological markers and HIV RNA levels, CD4 cell counts, plasma sCD14, or plasma LPS levels (data not shown). Thus, we found no association between IBD serological markers and HIV disease markers or plasma LPS levels in the study cohort.

**Table 3:** Clinical and serological profile in AIDS subjects having a serological pattern consistent with IBD versus not consistent with IBD.

	Pattern consistent with IBD**(n = 12)	Pattern not consistent with IBD**(n = 14)	p-value
Age (years)	45	44	0.149
Plasma HIV RNA (copies/ml)	9,725	10,935	0.665
CD4 cell count (cells/µl)	84	66	0.956
Plasma sCD14 (µg/ml)	2.5	2.6	0.897
Plasma LPS (pg/ml)	121	105	0.207
Plasma EndoCAb (MMU/ml)	57	75	0.738
ASCA IgA (EU/ml)	19.7	12	0.135
ASCA IgG (EU/ml)	31.4	12	0.027*
Anti-OmpC IgA (EU/ml)	25.9	7.1	0.005*
Anti- CBir1 (EU/ml)	18.6	9.1	0.001*
pANCA AutoAb (EU/ml)	12.1	12.1	0.540

Abbreviations: IBD - Inflammatory bowel disease;

**numbers represent median values; Statistical analysis was performed using Mann-Whitney test,

*p<0.05 statistically significant; ASCA IgA/IgG- Anti-Saccharomyces cerevisiae antibodies; Anti-OmpC IgA- Anti-Outer membrane porin C on E.coli; Anti-CBir1- recognizes bacterial flagellin antigen associated with IBD; pANCA autoantibody-

IBD-specific pANCA autoantibody (NSNA, Neutrophil-specific nuclear autoantibody).

### No association of IBD serological markers with plasma cytokines and chemokines in AIDS patients

In IBD, the dysregulated inflammatory response is associated with upregulation of mucosal and systemic levels of cytokines (i.e., IL-1, IL-6, IL-8, IL-12, and TNF) [73]. We used a multiplex assay to measure levels of 25 cytokines and chemokines in plasma samples from 20 AIDS patients in the study cohort and explore their relationship to individual IBD serological markers. We detected higher levels of IL-2R, CXCL9, CXCL10, CCL2, and IL-6 (p = 0.001, 0.0003, 0.0002, 0.017, and 0.0005, respectively) in plasma of AIDS patients compared to healthy uninfected controls (data not shown). No significant differences in cytokine/chemokine levels were seen when AIDS subjects were grouped according to positive (n = 9) versus negative IBD (n = 11) pattern (data not shown). Only IL-10 showed a trend towards significance (median 7.26 (range 0–41.94 pg/ml) versus 2.14 (0–8 pg/ml) respectively, p = 0.087, data not shown), with higher levels in patients with a positive IBD pattern. We then examined the association of individual IBD serological markers with levels of IL-1β, IL-2R, IL-6, IL-10, IL-17, IP-10, IFNγ, TNF, MIP-1α, MIP-1β, and MCP-1, and found a positive correlation only between anti-CBir1 antibody and IL-6 (r = 0.447, p = 0.048, Figure S2). Together with previous studies demonstrating that CBir1 antigen can induce IL-6 and other proinflammatory cytokines [56], [58], this finding raises the possibility that translocation of flagellin into the circulation may be a factor that contributes to immune activation in AIDS.

### Magnitude of IBD serological antibody response in AIDS

Previous data suggested that the magnitude of antibody responses to microbial antigens is associated with increased risk of complicated CD [25], [42], [59]. We tested this hypothesis with respect to HIV disease progression by counting the number of positive antibodies (ASCA, pANCA, anti-OmpC, and anti-CBir1) and scoring these from 0–3 (Table 4). None of the subjects showed a positive response for all 4 antibodies; 26.9% (7/26) had a score of 1, 30.7% (8/26) had a score of 2, 7.6% (2/26) had a score of 3, and 34.6% (9/26) had a score of zero. Forty-two percent (6/14) of subjects with CD4 T - cell counts <100 cells/µl versus 27% (3/11) with >100 cells/µl had antibody responses to 2 antigens, but this difference did not reach statistical significance (p = 0.676). Subjects grouped according to HIV RNA levels >10,000 versus <10,000 copies/ml, heroin use versus no heroin use, HAD versus non-HAD, or LPS > median value of 109 pg/ml versus <109 pg/ml had no difference in the magnitude of antibody response. Thus, the magnitude of IBD serological antibody responses was not associated with HIV disease markers, high LPS levels, or clinical subgroups in the study cohort.

**Table 4:** Clinical characteristics of AIDS patients in relation to the magnitude of IBD serological antibody response.

Subjects	Number of IBD serological antibodiesn (%)			
	0	1	2	3
AIDS patients (n = 26)	9 (34.6)	7 (26.9)	8 (30.7)	2 (7.6)
CD4 <100 cells/µl (n = 14)	4 (28.5)	3 (21.4)	6 (42.8)	1 (7.1)
Plasma HIV RNA >10,000 HIV RNA copies/ml (n = 12)	3 (25.0)	3 (25.0)	5 (41.6)	1 (8.3)
LPS >109 pg/ml* (n = 13)	4 (30.7)	4 (30.7)	4 (30.7)	1 (7.6)
Heroin IVDU (n = 13)	3 (23.0)	6 (46.1)	3 (23.0)	1 (7.6)
HAD (n = 13)	4 (30.7)	4 (30.7)	4 (30.7)	1 (7.6)

Abbreviations used: HAD- HIV-associated dementia; Heroin IVDU- heroin intravenous drug users; LPS- Lipopolysaccharide;

Antibodies tested- ASCA IgA/IgG- Anti-Saccharomyces cerevisiae antibodies; Anti-OmpC IgA- Anti-Outer membrane porin C on E.coli;

Anti-CBir1- recognizes bacterial flagellin antigen associated with IBD; pANCA autoantiody- IBD-specific pANCA autoantibody (NSNA, Neutrophil-specific nuclear autoantibody);

*represents cut-off above the median value for the study cohort.

## Discussion

In this study, we examined IBD serological markers in AIDS patients with high plasma LPS levels and detected an IBD-like serological pattern in 46%. Among subjects with a positive IBD pattern, 75% had a CD-like pattern and 25% had a UC-like pattern. The association of pANCA with CD markers has been described as a UC-like Crohn's pattern [25], [29], [43], [69], [70]. Based on this classification, 15% of the study cohort had a UC-like CD pattern. These findings are consistent with the frequent occurrence of HIV-related gut disease involving the small and large intestine in AIDS patients [1], [2], [3], [4]. Antibodies to microbial antigens (ASCA IgG, anti-OmpC, and anti-CBir1) were detected at higher levels in AIDS patients with compared to those without an IBD serological pattern. These findings together with the detection IBD serological markers in approximately 65% of AIDS patients with high plasma LPS suggest that IBD markers, in particular ASCA, anti-OmpC, and anti-CBir1, may provide a non-invasive approach to monitor HIV-related inflammatory gut disease, and possibly therapeutic responses.

Our original hypothesis was that high plasma LPS levels or HIV disease markers would be associated with detection of IBD serological markers. However, we found no difference in LPS levels between AIDS subjects with versus without an IBD-like serological pattern, and no association between the frequency or magnitude of IBD serological markers and HIV RNA levels, CD4 cell counts, plasma sCD14 or LPS levels. We found a higher magnitude of antibody responses to 2 antigens in AIDS patients with low CD4 counts or high viral loads as compared to other clinical subgroups, but this difference did not reach statistical significance. pANCA antibodies were previously detected in 18% to 41.9% of patients with symptomatic HIV infection [74], [75], [76], and in 20% of heroin users with systemic complications irrespective of HIV infection [77]. Consistent with these findings, we detected pANCA in 31% of AIDS patients and 38% of heroin users. Nonetheless, detection or levels of pANCA did not discriminate between subgroups classified according to positive IBD pattern, HIV disease markers, or IV heroin use. Together, these unexpected findings could reflect differences in gut commensalism, other host factors that affect gut homeostasis, or limitations of our study such as the small sample size, cross-sectional design, or selection of subjects with high LPS levels. Alternatively, the severity of HIV- related gut disease may not be detected by serological measurements of IBD markers or HIV disease biomarkers. We also cannot exclude the possibility that the absence of antibody responses to microbial antigens in 35% (9/26) of AIDS patients with high LPS levels reflects weakened humoral immune responses. Another limitation of our study is the lack of endoscopy or gut pathology data for the study cohort. Viazis et al [66] examined IBD outcomes in HIV-infected subjects who had an IBD diagnosis and found that these patients have a better disease course with lower probability of IBD relapse as compared to HIV-negative IBD patients. This was attributed to lower CD4 T-cell counts in HIV-infected individuals suppressing disease activity in CD. If true, low CD4 counts in our study cohort might influence relationships between HIV disease markers or LPS levels and IBD serological antibodies. The Th17 subset of CD4+ T-cells play an important role in the pathogenesis of IBD; HIV preferentially infects and depletes these cells in GALT, which may also help to explain the better disease course observed in patients with an IBD diagnosis who are HIV-positive compared to those who are HIV-negative. Larger prospective studies are needed to determine the clinical significance of IBD serological markers in HIV-infected patients and their relationship to HIV-related gut disease.

To assess the diagnostic precision of IBD serological antibodies and their ability to distinguish between CD, UC, inflammatory and non-inflammatory non-IBD disease subjects, and healthy controls, we performed meta-analysis of serological data from 20 published studies [25], [27]–[30], [33]–[35], [37], [42], [49], [52], [53], [55], [57], [58], [78]–[81]. Extensive meta-analysis was previously reported for ASCA and pANCA antibodies [43]. To our knowledge, however, the present study is the first meta-analysis for all available IBD serological markers. Consistent with previous reports [25], [27], [29], [30], [34], [37], [79], [81], our meta-analysis demonstrated that the prevalence of ASCA IgA/IgG was 45% in CD subjects as compared to 7.9% and 3.7% in non-IBD and healthy controls, respectively, and pANCA was prevalent in 42.4% of UC subjects (Table 5) [26], [27], [29], [32], [37], [44], [82]. Anti-OmpC and anti-CBir1 were prevalent in 29.4% and 55.2% of CD subjects, respectively, compared to 17.6% and 25.5% in non-IBD disease controls, and 10.7% and 6.3% in healthy controls. A separate meta-analysis of 10 published studies that used Prometheus Laboratories IBD Serological Markers (Table S1) demonstrated similar findings, with ASCA, anti-OmpC, and anti-CBir1 more prevalent in CD (48.5%, 32.2%, and 55.8%, respectively), pANCA more prevalent in UC (67.9%), and ASCA, pANCA, and anti-OmpC prevalent in 1.6%–6.3% of non-IBD disease controls and 3.9%–9.9% of healthy controls (Table S1). We detected ASCA, anti-OmpC, and anti-CBir1 in 15%–31% of AIDS patients with high LPS levels compared to 29.4%–55.2% in CD, 7.9%–25.5% in non-IBD disease controls, and 3.7%–0.7% in healthy controls. Thus, IBD serological markers were detected more frequently in AIDS patients than in non-IBD disease controls or healthy subjects, but less frequently than in CD patients.

**Table 5:** Meta-analysis of 20 studies using IBD serological markers.

Marker	Number of studies				Number of subjects				Positive for antibodies n (%)			
	CD	UC	Disease ontrols	Healthy ontrols	CD	UC	Disease controls	Healthy ontrols	CD	UC	Disease Controls	Healthy Controls
ASCA IgA/IgG*	15	9	6	6	4893	1026	455	1071	2206 (45)	110 (10.7)	36 (7.9)	40 (3.7)
ANCA	10	8	6	6	2424	826	381	871	352 (14)	351 (42.4)	54 (16)	23 (2.6)
Anti-OmpC	10	4	2	3	4116	448	176	401	1211 (29.4)	90 (20)	31 (17.6)	43 (10.7)
Anti-CBir1	6	2	2	2	2261	100	43	80	1249 (55.2)	11 (11)	11 (25.5)	5 (6.3)

CD- Crohn's Disease; UC- Ulcerative colitis;

*- any one of the antibodies present; Disease controls include inflammatory and non-inflammatory non-IBD disease controls (n = 633), including 264 non-IBD inflammatory gut diseases (i.e., colitis, gastroenteritis, celiac disease, etc); 193 non-inflammatory gut diseases (i.e., abdominal pain, diarrhea, lactose intolerance, etc); 90 rheumatologic disorders, and 86 other (i.e., constipation, nausea, rectal bleeding, etc).

The meta-analysis includes 19 studies with CD patients [25], [27], [28], [29], [30], [33], [34], [35], [37], [42], [49], [52], [53], [55], [57], [58], [78], [79], [81], 12 with UC patients [25], [27], [29], [30], [34], [37], [42], [52], [57], [58], [78], [79], [81], 10 with non-IBD disease controls [25], [29], [30], [37], [52], [57], [58], [78], [79], [80], [81], and 8 with healthy controls [25], [27], [29], [30], [34], [42], [57], [58], [81].

IBD serological markers alone are not recommended for diagnosis and monitoring of IBD because they are not robust enough for routine use [50], [83]. Moreover, prediction of IBD using Smart Diagnostic Algorithm Technology has not been published in peer-reviewed journals and test validation was done using only healthy controls [83]. To our knowledge, there are no published prospective studies on IBD serological markers in predicting disease course in UC or CD. As such, prospective studies are needed to determine their prognostic significance. Nonetheless, our meta-analysis highlights the utility of IBD serological markers in discriminating between CD, UC, non-IBD controls, and healthy controls, and their potential use for further studies of gut disease in HIV infection.

Several cytokines with proinflammatory activities (IL-1, IL-6, IL-8, IL-12, and TNF-α) are upregulated in IBD, and are likely to play an important role in clinical and immunopathological manifestations of the disease [56], [73]. Plasma IL-10 concentrations are elevated in subjects with active CD and UC [84] or with UC [85]. Similarly, higher levels of IL-10 in AIDS patients with compared to those without an IBD-like pattern were suggested by a trend towards significance in the present study. Higher levels of IL-6 [56], [73] and negative correlation between IL-6 and anti-CBir1 were previously reported in CD subjects [56]. In contrast, we found a positive correlation between IL-6 and anti-CBir1 in AIDS patients. These findings suggest complex relationships between antibody responses to CBir1 antigen and IL-6 induction, and raise the possibility that translocation of flagellin from the gut into the circulation, or associated pathogenic processes, might contribute to immune activation in chronic HIV infection.

Limitations of this study are its cross-sectional design and small sample size, which may have decreased the power to detect significant associations between IBD serological markers and HIV disease markers. Another limitation is the narrow selection criteria used to define the study cohort, limited to AIDS subjects with CD4 counts <300 cells/µl and high plasma LPS levels. These narrow selection criteria may in part explain our inability to detect a significant difference in HIV disease markers, plasma LPS levels, or plasma cytokine/chemokine levels between patients with and without an IBD-like pattern. In view of these limitations, we recognize the need for further studies to examine the detection of IBD serological markers and frequency of an IBD-like pattern in patients with acute or chronic HIV infection before progression to AIDS and in relation to gut disease documented by endoscopic or pathologic exam. Despite these limitations, this study opens the door for new opportunities to explore and validate the detection of IBD serological markers in peripheral blood samples as novel markers of gut disease in HIV infection.

In summary, we detected at least one IBD serological marker in approximately 65% of AIDS patients with high LPS levels, and an IBD-like serological pattern in 46%. Detection of these markers, particularly ASCA, anti-OmpC, and anti-CBir1, could provide a potential non-invasive approach to monitor HIV-related gut disease. Further studies are warranted to understand the clinical significance of IBD serological markers in HIV infection and their utility as tools for studies of HIV-related gut disease and monitoring therapeutic responses.

## Supporting Information

Figure S1 Differences in levels of IBD serological markers (A-ASCA IgA, B- ASCA IgG, C - Anti-OmpC and D- Anti-CBir1) in subjects with versus without IBD pattern are shown. Lines indicate median levels for each group. Mann Whitney test was used to assess the difference in antibody levels between the two groups, *p<0.05 was considered statistically significant. (EPS)

Figure S2 IL-6 levels correlated positively with levels of anti-CBir1 (n = 20 AIDS patients, Spearman correlation). (EPS)

Table S1 Meta-analysis of 10 studies using Prometheus laboratories IBD Serology 7 (DOC)
