# Supplementary material for: Serological Markers for Inflammatory Bowel Disease in AIDS Patients with Evidence of Microbial Translocation
Source: PLoS One. 2010 Nov 15;5(11):e15533. doi: 10.1371/journal.pone.0015533 (PMC2981579; doi:10.1371/journal.pone.0015533)
Supplement: Table S1 — Meta-analysis of 10 studies using Prometheus laboratories IBD Serology 7 (DOC) [file pone.0015533.s003.doc]

**Table S1.** Meta-analysis of 10 studies using Prometheus laboratories IBD Serology 7

| **Marker** | **Number of studies** | | | | **Number of subjects** | | | | **Positive for antibodies n (%)** | | | |
| --- | --- | --- | --- | --- | --- | --- | --- | --- | --- | --- | --- | --- |
|  | **CD** | **UC** | **Disease**  **Controls** | **Healthy**  **Controls** | **CD** | **UC** | **Disease**  **Controls** | **Healthy**  **Controls** | **CD** | **UC** | **Disease**  **Controls** | **Healthy**  **Controls** |
| ASCA IgA/IgG* | 8 | 3 | 2 | 2 | 2603 | 181 | 141 | 201 | 1265 (48.5) | 9 (4.9) | 9 (6.3) | 8 (3.9) |
| ANCA | 4 | 2 | 1 | 1 | 1147 | 81 | 63 | 101 | 207 (18) | 55 (67.9) | 1 (1.6) | 7 (6.9) |
| Anti-OmpC | 7 | 2 | 1 | 1 | 2503 | 81 | 63 | 101 | 806 (32.2) | 14 (17.2) | 3 (4.8) | 10 (9.9) |
| Anti-CBir1** | 4 | - | - | - | 2061 | - | - | - | 1151 (55.8) | - | - | - |

*CD- Crohn’s Disease; UC- Ulcerative colitis; * - any one of the antibodies present; ** there are no published studies using Prometheus IBD Serology for detection of anti-CBir in non-IBD and healthy controls. Disease controls include inflammatory and non-inflammatory non-IBD disease controls (n=267), including 98 non-IBD inflammatory gut diseases (i.e., colitis, gastroenteritis, celiac disease, etc); 132 non-inflammatory gut diseases (i.e., abdominal pain, diarrhea, lactose intolerance, etc); 2 rheumatologic disorders, and 35 other (i.e., constipation, nausea, rectal bleeding, etc). The meta-analysis includes 10 studies with CD patients [28,34,35,37,49,52,53,55,78,81], 5 with UC patients [34,37,52,78,81], 4 with non-IBD disease controls [37,52,78,81], and 2 with healthy controls [34,81].*
